# Causal effects of blood cells on breast cancer: Evidence from bidirectional Mendelian randomization combined with meta-analysis

**DOI:** 10.1097/MD.0000000000041545

**Published:** 2025-02-14

**Authors:** Qi Liu, Wei Jia, Yi Zhang, Jun Lu, Qingbin Luo, Lin Yang, Dongdong Wan

**Affiliations:** a Department of Oncology, Anhui Zhongke Gengjiu Hospital, Hefei, Anhui Province, China; b Department of Medical Oncology, The First Affiliated Hospital of USTC, Hefei, Anhui Province, China; c Department of Medical Oncology, Nantong Haimen District People’s Hospital, Nantong, Jiangsu Province, China.

**Keywords:** bidirectional Mendelian randomization analysis, blood cell, breast cancer, meta analysis, multiple corrections

## Abstract

Recent studies suggest blood cells influence breast cancer, but no Mendelian randomization (MR) studies have confirmed a causal relationship between specific blood cell phenotypes and breast cancer. MR analysis of blood cell phenotypes used breast cancer data from Finngen R11, UKB, and open genome-wide association study databases. Meta-analyzed inverse variance weighted results were adjusted for multiple comparisons. The reverse relationship was also explored. MR and meta-analysis identified significant associations between specific blood cell phenotypes and breast cancer: neutrophil perturbation response (side fluorescence standard deviation of neutrophil 4 in response to alhydrogel perturbation): odds ratio (OR) = 0.967, *P* = .0009; neutrophil perturbation response (forward scatter median of neutrophil 4 in response to Pam3CSK4 perturbation): OR = 0.972, *P* = .031; white blood cell perturbation response (side scatter coefficient of variation of WBC 2 in response to nigericin perturbation): OR = 0.972, *P* = .031; white blood cell perturbation response (forward scatter coefficient of variation of WBC in response to Pam3CSK4 perturbation): OR = 1.042, *P* = 8.15 × 10^−5^. And there was no reverse result. Neutrophil perturbation response (side fluorescence standard deviation of neutrophil 4 in response to alhydrogel perturbation) and white blood cell perturbation response (side scatter coefficient of variation of WBC 2 in response to nigericin perturbation) are protective factors for breast cancer. Conversely, neutrophil perturbation response (forward scatter median of neutrophil 4 in response to Pam3CSK4 perturbation) and white blood cell perturbation response (forward scatter coefficient of variation of WBC in response to Pam3CSK4 perturbation) are risk factors for breast cancer.

## 1. Introduction

Breast cancer is the most prevalent cancer among women globally, with around 2 million new cases diagnosed each year. It ranks as a leading cause of cancer-related deaths in women, claiming about 600,000 lives annually. Several factors influence the risk of developing breast cancer, including uncontrollable elements such as gender (women are at higher risk), age (risk increases after 50), family history (particularly with first-degree relatives), genetic mutations (like BRCA1 and BRCA2), early onset of menstruation, and late menopause. Modifiable risk factors include prolonged use of hormone replacement therapy (HRT), heavy alcohol consumption, obesity, physical inactivity, and delayed or no childbirth. Symptoms typically involve the presence of lumps in the breast, alterations in shape or size, skin changes, nipple modifications, and swollen lymph nodes in the armpit area.^[1,2]^

Globally, breast cancer incidence has been on the rise, with approximately 2 million new cases and 600,000 deaths reported each year. The global 5-year survival rate is generally around 80% to 85%, though this varies by region and healthcare access. Early-stage breast cancer often does not present clear symptoms, so regular screening methods such as mammograms, clinical examinations, and self-checks are critical for early detection and improving outcomes through timely intervention.^[3]^ With advancements in medical technology, treatment methods, including surgery, radiotherapy, chemotherapy, and targeted therapy, have gradually improved the cure and survival rates for breast cancer.

Existing research on white blood cells in breast cancer primarily focuses on their counts and proportions in relation to disease prognosis. Studies indicate that an increase in the total white blood cell count in breast cancer patients may be associated with disease progression and metastasis risk. Specifically, the lymphocyte-to-monocyte ratio (LMR) is considered a potential prognostic marker, with low LMR reflecting an immunosuppressive state and associated with poor prognosis. Changes in LMR may affect patient response to treatment, with low LMR potentially indicating poorer tolerance to chemotherapy and radiotherapy. Additionally, higher levels of tumor-infiltrating lymphocytes in the tumor microenvironment are generally associated with better prognosis, especially in triple-negative breast cancer. High levels of tumor-infiltrating lymphocytes can not only directly kill tumor cells but also regulate the tumor microenvironment and enhance immune responses by releasing various cytokines.^[4,5]^

The role of neutrophils in breast cancer is mainly reflected in the neutrophil-to-lymphocyte ratio (NLR) and their function in the tumor microenvironment. High NLR is considered an independent predictor of poor prognosis in breast cancer patients, reflecting systemic inflammation and immunosuppressive states. High NLR may be associated with higher tumor invasiveness and metastatic potential, as neutrophils promote tumor cell growth and metastasis by secreting cytokines and proteases and releasing neutrophil extracellular traps that further promote cancer cell metastasis. Moreover, neutrophils may help tumor cells escape immune surveillance by inhibiting the function of anti-tumor T cells. Studies also suggest that changes in neutrophil activity and function may be related to different subtypes of breast cancer, with certain subtypes being more sensitive to neutrophil activity. Targeted therapies against neutrophils are becoming a research focus, aiming to inhibit their pro-tumor effects and enhance anti-tumor immune responses. Future research may further elucidate the specific mechanisms of neutrophils in breast cancer and explore how to leverage these mechanisms for new therapeutic interventions.^[6,7]^

Mendelian randomization (MR) is an epidemiological approach that uses genetic variants as proxies to explore causal relationships between exposures and outcomes, offering several key benefits. First, by utilizing the random inheritance of genes, MR minimizes the influence of confounding factors, such as socioeconomic status. Second, it helps mitigate reverse causality, since genetic variants are established before the exposure and outcome occur. Furthermore, MR can assess the long-term effects of exposures, unlike studies that focus on short-term correlations. Its noninvasive nature also makes it more ethically acceptable, particularly when randomized controlled trials (RCTs) are impractical or unfeasible. With the use of large-scale genomic data, MR studies gain increased statistical power and the ability to apply sophisticated techniques to untangle complex causal relationships. In summary, MR offers significant advantages in minimizing bias and strengthening the reliability of causal conclusions.

This study employed bidirectional MR alongside meta-analysis to explore the causal connections between 91 distinct blood cell phenotypes and breast cancer, highlighting its notable strengths. Bidirectional MR not only assessed the potential causal impact of blood cell traits on breast cancer but also investigated how breast cancer might influence these phenotypes, offering a comprehensive view of the 2-way causal relationships. Additionally, by combining data from multiple studies through meta-analysis, the research increased statistical power and reinforced the reliability of the findings. This approach presents an innovative framework for investigating the potential mechanisms driving breast cancer and lays a solid foundation for advancing its treatment and prevention strategies.

## 2. Methods and materials

### 2.1. Study design

This study employed a sophisticated triangulation methodology that integrated bidirectional MR and meta-analysis to thoroughly investigate the causal links between 91 distinct blood cell phenotypes and breast cancer. The first step involved the collection and preprocessing of both exposure and outcome data. Specifically, exposure data related to the 91 blood cell phenotypes were carefully prepared for analysis. The MR analysis was then performed independently using breast cancer data sourced from 3 separate and distinct databases, ensuring that the data for the blood cell phenotypes (the exposures) and the breast cancer outcomes came from independent sources. This approach helped to minimize potential biases that might arise if the same datasets were used for both the exposure and outcome variables.

Following the initial MR analysis, the inverse variance weighted (IVW) method was applied to aggregate the MR results for each blood cell phenotype across the 3 breast cancer datasets. These individual MR results were then synthesized through a meta-analysis, pooling findings from the 3 datasets. To enhance the precision and validity of the meta-analysis, the significance P-values obtained were adjusted for multiple comparisons, reducing the likelihood of Type I errors and ensuring the robustness of the results.

By synthesizing data from various large-scale studies, the research was able to offer a more comprehensive and reliable evaluation of the causal relationship between blood cell phenotypes and breast cancer. This method not only provided deeper insights into the research question but also contributed to more credible and statistically robust conclusions. In addition to exploring direct causal pathways, the study also took an important step in validating the causal direction. Reverse causality tests were conducted on the significant blood cell phenotype data to determine whether breast cancer could influence blood cell phenotypes, ensuring that the observed associations were truly causal and not the result of reverse causation. This dual approach (forward and reverse causality) strengthens the credibility of the causal inferences made in the study.^[8]^

A recent study applied the MR approach to explore the causal relationship between circulating antioxidant levels and the risk of coronary heart disease (CHD). Using genetic instrumental variables and CHD outcome data from 3 separate databases, researchers assessed the potential link between antioxidants and CHD. The IVW method was employed to calculate effect sizes for each database, with careful validation of the genetic instruments to ensure the reliability and independence of the data. Following this, the IVW results from all 3 databases were combined through meta-analysis, which boosted statistical power and reduced uncertainty in the findings. The comprehensive analysis ultimately revealed no substantial evidence supporting a protective effect of circulating antioxidants against CHD, both in individual database assessments and in the meta-analysis. This result challenges the widely held belief that antioxidants may lower cardiovascular disease risk, suggesting that they might not be effective as protective factors against CHD.^[8]^

**Figure 1.**
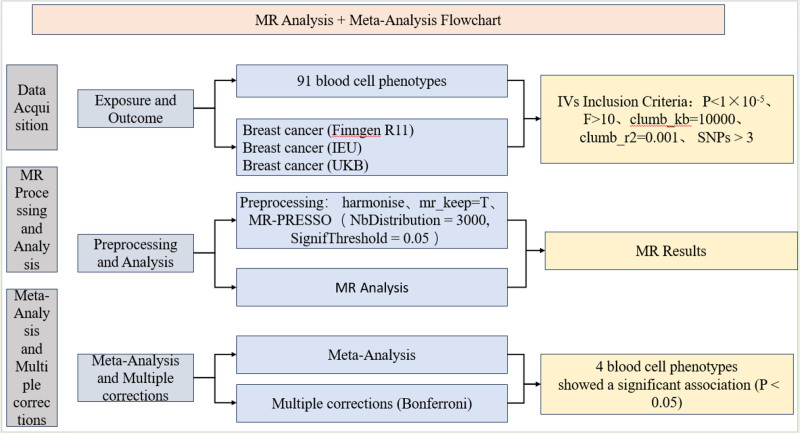
The process flowchart of the research methodology. IV = instrumental variable, MR = Mendelian randomization, SNP = single nucleotide polymorphism.

It is worth mentioning that this study involved the processing and analysis of publicly available data, thereby exempting it from further ethical review. However, the ethical approval of the original data is included in the ethics statement section. Additionally, to clearly illustrate the research process, relevant flowcharts have been completed (Fig. 1).

### 2.2. Genome-wide association study (GWAS) data sources for the 91 types of blood cells

This study retrieved exposure data from the publicly available genome-wide association study (GWAS) catalog. The original investigation examined how genetic variations influence the behavior of human blood cells under different stress conditions. Flow cytometry was used to analyze blood cells from more than 4700 individuals exposed to 37 distinct perturbation scenarios, identifying 119 genomic loci and 96 genes linked to specific cell phenotypes. These phenotypes are connected to various common diseases, such as a pro-inflammatory and anti-apoptotic neutrophil population found in cardiometabolic conditions. The study’s results suggest that this methodology could improve disease classification and enable more personalized treatments, advancing the field of precision medicine.

The data from the original study on how genetic variations affect human blood cell responses under different stress conditions involved 91 blood cell phenotypes, and the GWAS codes for these immune phenotypes were GCST90257015-GCST90257105.^[9]^ Based on recent research, we filtered the data according to the following criteria: *P* < 1 × 10^−5^, *F* > 10, clump_kb = 10,000, and clump_*r*2 = 0.001. The final number of single nucleotide polymorphisms (SNPs) that met these criteria was 887.

### 2.3. Sources of GWAS data on breast cancer

The outcome data for this study was primarily sourced from 3 databases different from the exposure data sources: the Open GWAS database, the UKB database, and the Finngen R11 database.

1. Open GWAS database: The breast cancer data in this database belongs to the Breast Cancer Association Consortium (BCAC), with the specific GWAS identifier ieu-a-1126.^[10]^ The data includes a total of 228,951 individuals, with 122,977 cases and 105,974 controls, encompassing 10,680,257 SNPs. [Download link] (https://gwas.mrcieu.ac.uk/files/ieu-a-1126/ieu-a-1126.vcf.gz).
2. Finngen R11 database: This database provides breast cancer data for a total of 222,080 individuals, with 20,586 cases and 201,494 controls, covering 21,297,594 SNPs.^[11]^ [Download link] (https://storage.googleapis.com/finngen-public-data-r11/summary_stats/finngen_R11_C3_BREAST_EXALLC.gz).
3. UKB database: The breast cancer data from this database includes a total of 385,438 individuals, with 32,029 cases and 353,409 controls, covering 13,984,675 SNPs (Pan-UKB team, https://pan.ukbb.broadinstitute.org, 2020). [Download link] (https://pan-ukb-us-east-1.s3.amazonaws.com/sumstats_flat_files/categorical-20110-both_sexes-5.tsv.bgz).

The breast cancer data from these 3 outcome databases were sourced from different populations than the exposure data, thus avoiding data overlap and ensuring accuracy. The datasets are among the best in their respective databases, with sufficient sample sizes and SNP counts to ensure the generalizability of the study results.

### 2.4. Criteria for selection of instrumental variables (IVs)

In MR studies, selecting appropriate instrumental variables (IVs) is essential. To ensure a strong connection with various blood cell traits, this study applied a *P* value cutoff of less than 1 × 10^−5^, including only single nucleotide polymorphisms (SNPs) that met this threshold. Additionally, each blood cell phenotype was required to have at least 3 SNPs linked to it, ensuring the data’s relevance and comprehensiveness. To refine the selection further, the *F* statistic for each SNP was calculated using the formula *F* = (beta/SE)^2^ (Table S1, Supplemental Digital Content, http://links.lww.com/MD/O396), with only SNPs having an *F* value >10 being retained. This step helped eliminate weak IVs, thus improving the robustness of the findings. Lastly, the data was prepared for MR analysis, and linkage disequilibrium was addressed to avoid its influence on the accuracy of the results. An linkage disequilibrium threshold of 0.001 and a distance cutoff of 10,000 kb were applied to ensure the independence of the SNPs, thereby enhancing the precision of the analysis.^[12,13]^

## 3. Statistical analysis

### 3.1. Causal validation between 91 types of blood cells and breast cancer

All data analyses were carried out using R version 4.2.1 (https://www.r-project.org/). Initially, SNPs associated with breast cancer were selected from 3 independent databases, distinct from the exposure data sources, and matched with SNPs in the exposure data, retaining only those that were common between both datasets. Palindromic SNPs were processed based on the action = 2 standard, and SNPs marked with mr_keep = false were excluded. Prior to performing MR-PRESSO, horizontal pleiotropy tests were conducted. SNPs with a *P* value <.05 in the pleiotropy test were considered horizontally pleiotropic and identified as outliers, which were then removed using the MR-PRESSO method (parameters: NbDistribution = 3000, SignifThreshold = 0.05). SNPs with *P* values >.05 were deemed not to exhibit pleiotropy and did not require further treatment.

For heterogeneity testing before the MR analysis, SNPs with significant heterogeneity (Q_pval < 0.05) were analyzed using the IVW random effects model, while those without significant heterogeneity were analyzed using the IVW fixed effects model. Regardless of heterogeneity, MR-Egger and weighted median methods were also employed, and odds ratios (ORs) were calculated. To enhance the robustness of the findings, MR analyses were performed for 91 blood cell phenotypes using 3 separate breast cancer datasets, followed by a meta-analysis of the IVW results from these analyses. *P* values from the meta-analysis were adjusted for multiple comparisons using the Bonferroni correction to reduce the risk of Type I errors. After this correction, 4 blood cell phenotypes were found to have significant associations with breast cancer.

### 3.2. Causal validation of the inverse relationship between positive blood cells and breast cancer

In this step, we utilized the significant blood cell phenotypes identified in the previous analysis as outcome variables, while the 3 breast cancer datasets served as the exposure variables. The same criteria for selecting instrumental variables and conducting data analysis, as applied in the forward analysis, were used here. The primary goal of this phase was to assess the direction of the causal relationship between the 2. The results provided no evidence supporting the presence of reverse causality between the blood cell phenotypes and breast cancer.

### 3.3. Sensitivity analysis

Horizontal pleiotropy refers to a scenario where a genetic variant affects multiple traits independently of the exposure of interest, which can lead to distorted conclusions about the causal relationship. To minimize the influence of horizontal pleiotropy on our results, we conducted pleiotropy tests on the GWAS data. SNPs showing significant horizontal pleiotropy (*P* value < .05) were identified as outliers and excluded using the MR-PRESSO method. The exclusion criteria were set with NbDistribution = 3000 and SignifThreshold = 0.05 to ensure that only robust data remained for further analysis^[14]^ (Table S2, Supplemental Digital Content, http://links.lww.com/MD/O396).

Heterogeneity refers to the variation or differences that exist among study subjects, observations, or experimental conditions. In research, heterogeneity typically reflects differences between samples or individuals, which could arise from factors such as genetic diversity, environmental influences, or socioeconomic conditions. While heterogeneity can enhance the generalizability and representativeness of research findings, it also adds complexity and can complicate interpretation. In this study, we conducted heterogeneity tests on the data as part of the analysis process. For SNPs showing significant heterogeneity (Q_pval < 0.05), the IVW random effects model was applied in the MR analysis. Conversely, for SNPs without significant heterogeneity, we used the IVW fixed effects model to ensure the results remained accurate and reliable^[15]^ (Table S3, Supplemental Digital Content, http://links.lww.com/MD/O396).

## 4. Results

### 4.1. Causal validation between 91 types of blood cells and breast cancer

MR analyses were conducted on 91 blood cell phenotypes with 3 sets of breast cancer data (Table S4, Supplemental Digital Content, http://links.lww.com/MD/O396). The IVW results from these MR analyses were subjected to meta-analysis (Table S5, Supplemental Digital Content, http://links.lww.com/MD/O396), and the significance *P* values from the meta-analysis were corrected for multiple comparisons. Ultimately, only 4 blood cell phenotypes showed significant associations with breast cancer. Notably, 2 blood cell phenotypes (GCST90257024, GCST90257077) had *P* values < .05 after correction, but their β-value directions were inconsistent across several main methods, preventing their classification as positive results (Table S6, Supplemental Digital Content, http://links.lww.com/MD/O396).

**Figure 2.**
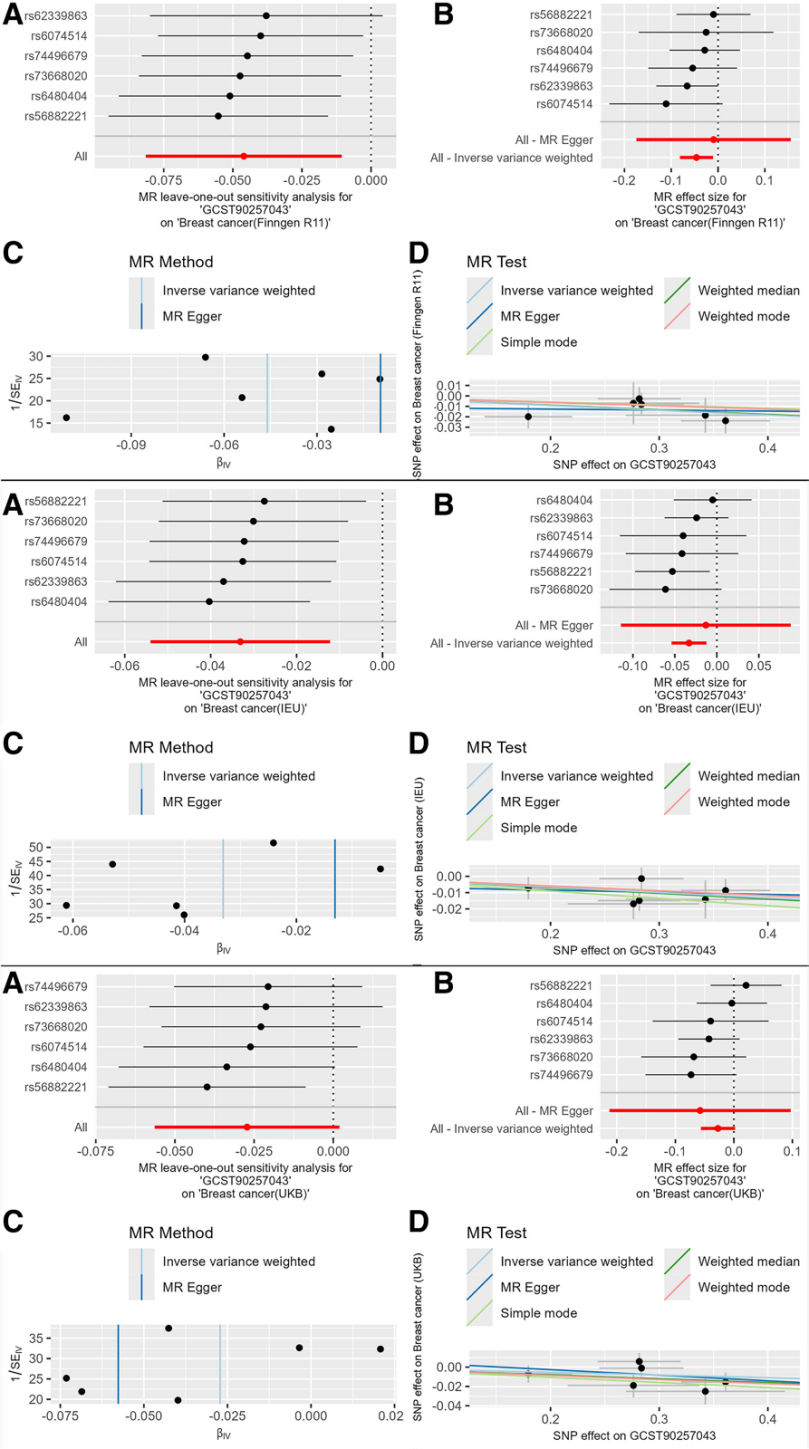
Combined MR plots of GCST90257043 on breast cancer. MR = Mendelian randomization, SNP = single nucleotide polymorphism.

Specifically, the blood cell phenotype Neutrophil perturbation response (side fluorescence standard deviation of neutrophil 4 in response to alhydrogel perturbation measured by WDF dye; GCST90257043) had the following results: Finngen database: The IVW result was OR = 0.955 (95% confidence interval [CI]: 0.922–0.989, *P* = .011), with consistent β value directions across 3 main methods, indicating it as a protective factor against breast cancer. UKB database: The IVW result was OR = 0.973 (95% CI: 0.947–1.000, *P* = .05), with consistent β value directions, still indicating a protective factor. Open GWAS database: The IVW result was OR = 0.967 (95% CI: 0.947–0.988, *P* = .002), with consistent β value directions, still indicating a protective factor. Meta-analysis result: The IVW result was OR = 0.967 (95% CI: 0.952–0.982, *P* = .0009), with significance corrected using the Bonferroni method (Table S6, Supplemental Digital Content, http://links.lww.com/MD/O396). Additionally, there were 6 strongly positive SNPs identified in this causal relationship. In addition, the blood cell phenotype and the MR combination diagram of breast cancer from 3 databases were also plotted (Fig. 2).

**Figure 3.**
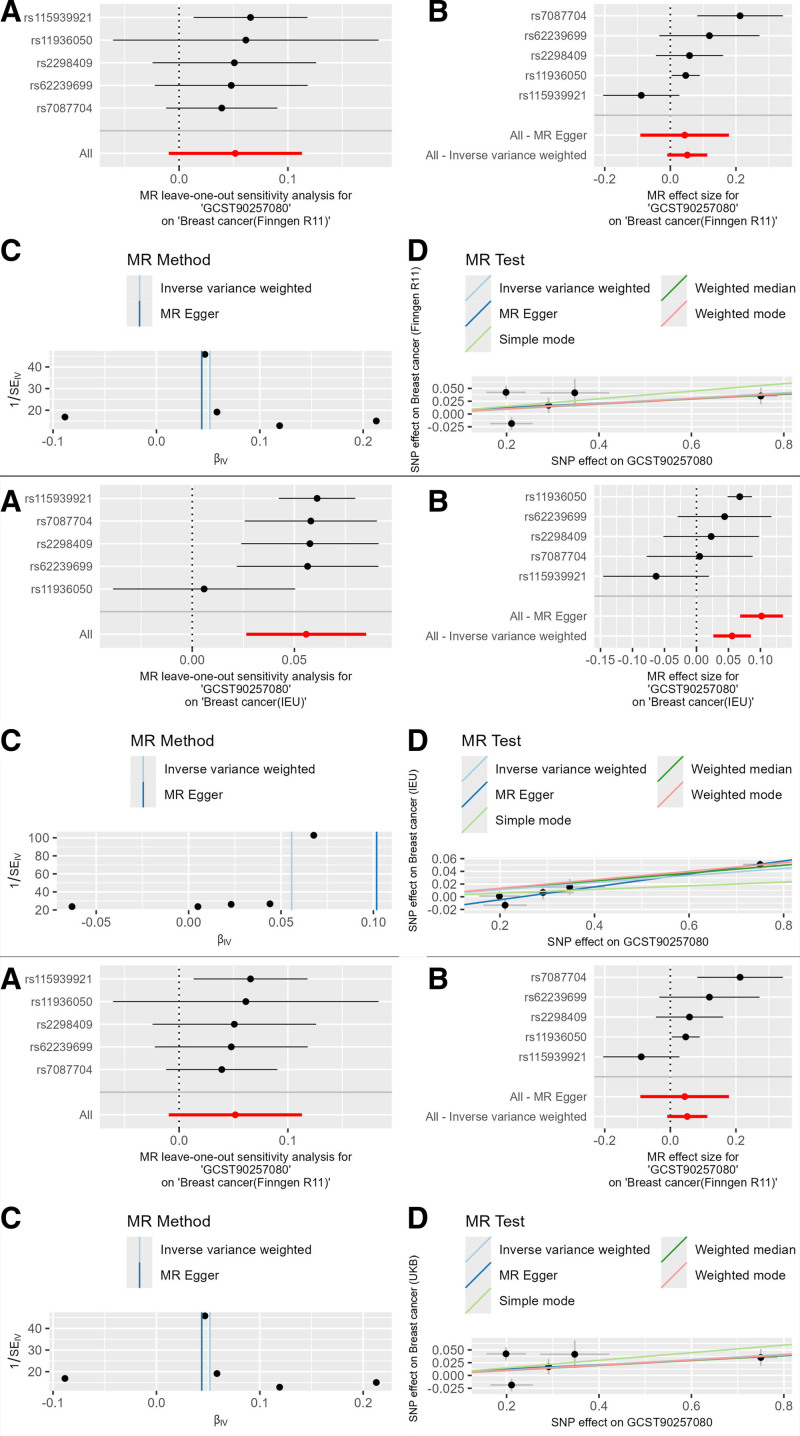
Combined MR plots of GCST90257080 on breast cancer. MR = Mendelian randomization, SNP = single nucleotide polymorphism.

The blood cell phenotype Neutrophil perturbation response (forward scatter median of neutrophil 4 in response to Pam3CSK4 perturbation measured by WDF dye; GCST90257080) had the following results: Finngen database: The IVW result was OR = 1.053 (95% CI: 0.991–1.120, *P* = .098), with consistent β value directions across 3 main methods, indicating it as a risk factor for breast cancer. UKB database: The IVW result was OR = 1.034 (95% CI: 0.993–1.077, *P* = .120), with consistent β value directions, still indicating a risk factor. Open GWAS database: The IVW result was OR = 1.057 (95% CI: 1.027–1.089, *P* = .0002), with consistent β value directions, still indicating a risk factor. Meta-analysis result: The IVW result was OR = 1.050 (95% CI: 1.027–1.073, *P* = .001), with significance corrected using the Bonferroni method (Table S6, Supplemental Digital Content, http://links.lww.com/MD/O396). There were 5 strongly positive SNPs identified in this causal relationship. And plotted the blood cell phenotype and the MR combination diagram of breast cancer from 3 databases (Fig. 3).

**Figure 4.**
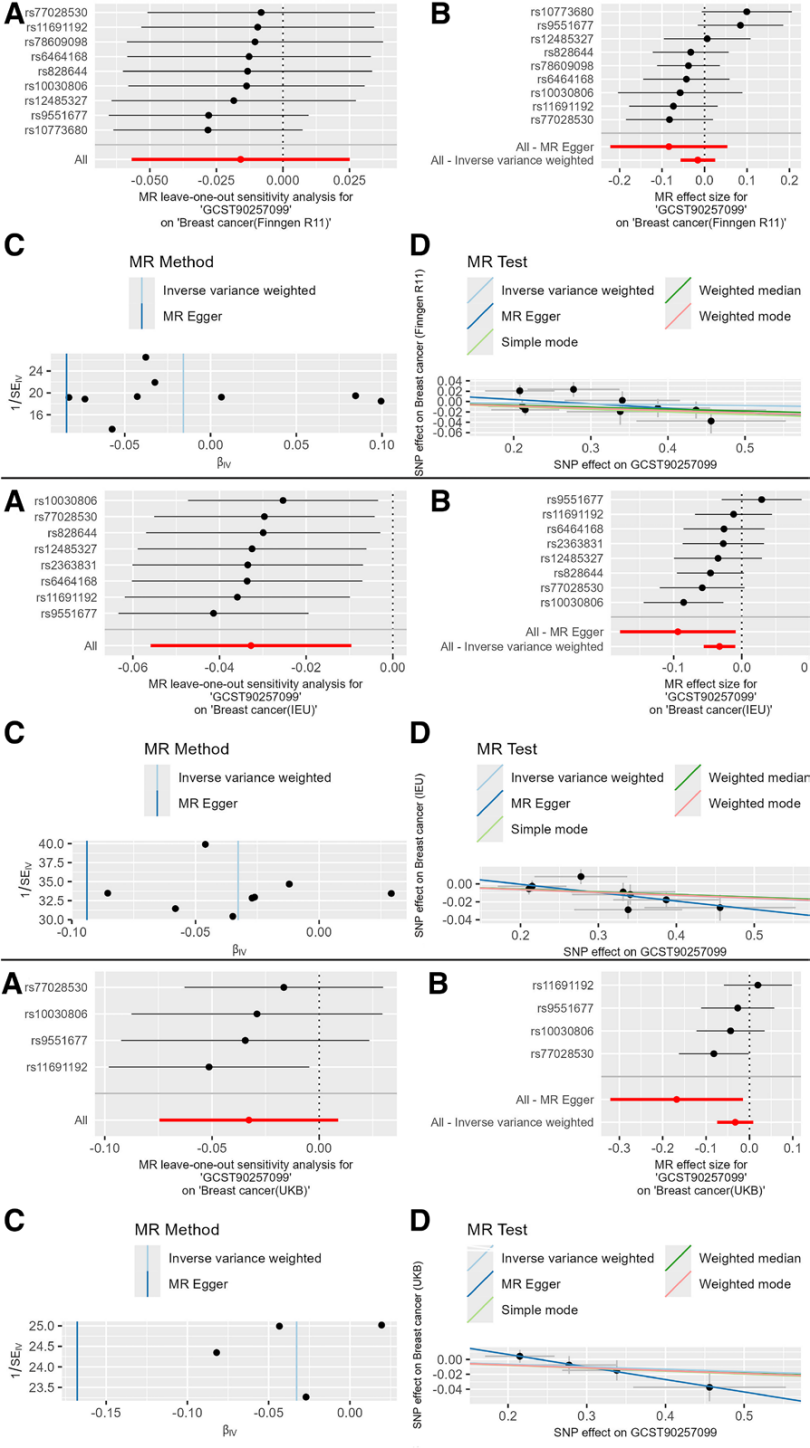
Combined MR plots of GCST90257099 on breast cancer. MR = Mendelian randomization, SNP = single nucleotide polymorphism.

The blood cell phenotype white blood cell perturbation response (side scatter coefficient of variation of WBC 2 in response to nigericin perturbation measured by WNR dye; GCST90257099) had the following results: Finngen database: The IVW result was OR = 0.984 (95% CI: 0.952–1.017, *P* = .344), with consistent β-value directions across 3 main methods, indicating it as a protective factor against breast cancer. UKB database: The IVW result was OR = 0.968 (95% CI: 0.930–1.007, *P* = .109), with consistent β-value directions, still indicating a protective factor. Open GWAS database: The IVW result was OR = 0.968 (95% CI: 0.948–0.988, *P* = .002), with consistent β-value directions, still indicating a protective factor. Meta-analysis result: The IVW result was OR = 0.972 (95% CI: 0.956–0.987, *P* = .031), with significance corrected using the Bonferroni method (Table S6, Supplemental Digital Content, http://links.lww.com/MD/O396). There were 9 strongly positive SNPs identified in this causal relationship. Similarly, a combination diagram was also plotted for the MR results (Fig. 4).

**Figure 5.**
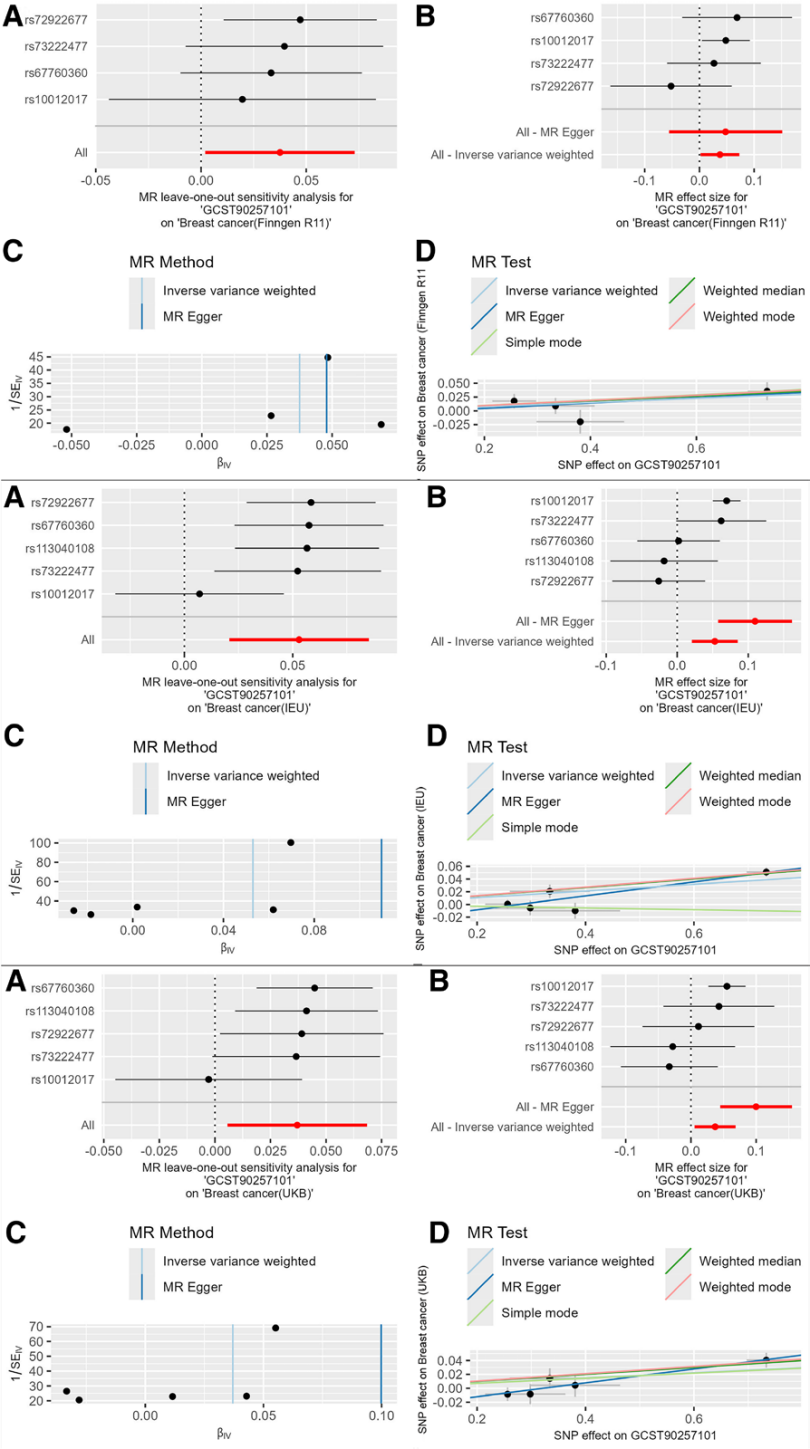
Combined MR plots of GCST90257101 on breast cancer. MR = Mendelian randomization, SNP = single nucleotide polymorphism.

The blood cell phenotype White blood cell perturbation response (forward scatter coefficient of variation of WBC in response to Pam3CSK4 perturbation measured by WNR dye; GCST90257101) had the following results: Finngen database: The IVW result was OR = 1.038 (95% CI: 1.002–1.074, *P* = .033), with consistent β-value directions across 3 main methods, indicating it as a risk factor for breast cancer. UKB database: The IVW result was OR = 1.038 (95% CI: 1.014–1.062, *P* = .002), with consistent β-value directions, still indicating a risk factor. Open GWAS database: The IVW result was OR = 1.054 (95% CI: 1.021–1.089, *P* = .001), with consistent *β*-value directions, still indicating a risk factor. Meta-analysis result: The IVW result was OR = 1.042 (95% CI: 1.025–1.060, *P* = 8.15 × 10^−5^), with significance corrected using the Bonferroni method (Table S6, Supplemental Digital Content, http://links.lww.com/MD/O396). There were 5 strongly positive SNPs identified in this causal relationship. Similarly, a combination diagram was also plotted for the MR results (Fig. 5).

**Figure 6.**
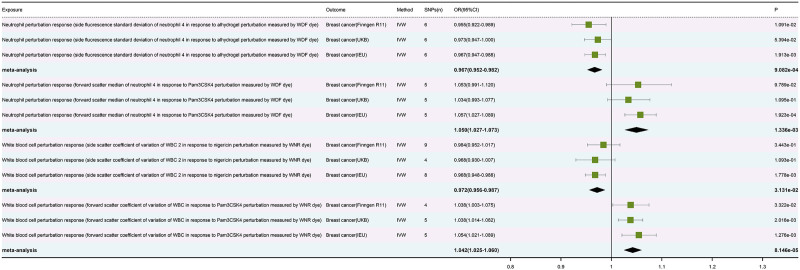
Forest plot of positive results after meta-analysis. IVW = inverse variance weighted.

In addition, it is worth mentioning that we also plotted forest plots for the meta-analysis and multiple correction results of the 4 highly significant blood cells mentioned above (Fig. 6).

### 4.2. Causal validation of the inverse relationship between positive blood cells and breast cancer

In the reverse validation process, we used the above 4 positive blood cell phenotypes as outcome data and breast cancer data from 3 different databases as exposure data. The data processing and instrumental variable selection were consistent with the forward analysis. In this process, we did not find significant associations between the 4 positive blood cell phenotypes and breast cancer across the different databases.

Specifically, when breast cancer data from the Finngen R11, UKB, and Open GWAS databases were used as exposure, and the blood cell phenotype neutrophil perturbation response (side fluorescence standard deviation of neutrophil 4 in response to alhydrogel perturbation measured by WDF dye; GCST90257043) was used as outcome, the MR analysis IVW results were *P* = .288, *P* = .265, and *P* = .714, respectively, indicating no causal relationship between this blood cell phenotype and breast cancer in any of the 3 databases.

For the blood cell phenotype neutrophil perturbation response (forward scatter median of neutrophil 4 in response to Pam3CSK4 perturbation measured by WDF dye; GCST90257080), the MR analysis results were *P* = .953, *P* = .646, and *P* = .139, respectively, again indicating no reverse causal relationship with breast cancer.

For the blood cell phenotype White blood cell perturbation response (side scatter coefficient of variation of WBC 2 in response to nigericin perturbation measured by WNR dye; GCST90257099), the MR analysis results were *P* = .409, *P* = .474, and *P* = .483, respectively, indicating no reverse causal relationship with breast cancer.

Finally, for the blood cell phenotype White blood cell perturbation response (forward scatter coefficient of variation of WBC in response to Pam3CSK4 perturbation measured by WNR dye; GCST90257101), the MR analysis results were *P* = .723, *P* = .259, and *P* = .584, respectively, indicating no reverse causal relationship with breast cancer in any of the databases (Table S7, Supplemental Digital Content, http://links.lww.com/MD/O396).

## 5. Discussion

Breast cancer is the most prevalent cancer among women worldwide, with approximately 2 million new diagnoses and 600,000 deaths each year. In developed nations, the incidence rate ranges from 70 to 100 cases per 100,000 people annually, whereas in developing countries, it is between 30 and 50 cases per 100,000 people. The risk of breast cancer rises significantly in women over the age of 50, with around 75% of cases occurring in this age group. Incidence rates vary across regions, being highest in North America and Europe, and lower in Asia and Africa. Genetic factors contribute to 5% to 10% of cases, and women with BRCA1 or BRCA2 mutations face a 50% to 85% lifetime risk of developing breast cancer. In developed countries, the 5-year survival rate is 85% to 90%, while in developing countries it is lower, at 60% to 70%. However, the survival rate for early-stage breast cancer detection can reach as high as 99%.^[16,17]^

Research into the relationship between blood cells and breast cancer highlights the critical roles of circulating tumor cells, immune cells, platelets, and white blood cells in the initiation, progression, and metastasis of the disease. Circulating tumor cells, as key markers of metastasis, are closely associated with prognosis, with both their quantity and characteristics providing valuable insights. Immune cells, particularly T cells and natural killer (NK) cells, play a central role in anti-tumor immunity, with ongoing studies investigating the enhancement of T cell function using immune checkpoint inhibitors. Platelet activation, which fosters tumor growth and angiogenesis, is also linked to poor prognosis, with elevated platelet counts correlating with worse outcomes. Furthermore, the NLR and monocyte levels in white blood cells are important factors in breast cancer progression and metastasis. Emerging biomarkers like circulating tumor DNA and exosomes hold promise for early detection and monitoring of the disease. Research suggests that the interactions between blood cells and the tumor microenvironment are intricate, involving various signaling pathways and gene expression modifications. Cytokines and chemokines secreted by blood cells influence not only tumor cell behavior but also other cell types within the tumor microenvironment, including fibroblasts and endothelial cells. Additionally, combining anti-platelet therapy with immunotherapy is gaining attention as a promising approach in breast cancer treatment.^[18–20]^ These studies highlight the complex roles of blood cells in the progression of breast cancer, offering new insights for the development of personalized treatment strategies and precision medicine. Ongoing research will delve deeper into these mechanisms to enhance treatment efficacy and improve patient outcomes.

An observational study involving over 1500 breast cancer patients explored the correlation between the NLR and patient prognosis. The findings revealed that patients with elevated NLR had significantly worse overall survival and disease-free survival. In particular, the 5-year survival rate for patients with high NLR (NLR ≥ 3) was substantially lower compared to those with low NLR (NLR < 3). Even after adjusting for potential confounders, including age, tumor size, lymph node involvement, and hormone receptor status, NLR remained a significant independent prognostic factor. The study suggested that a high NLR could indicate a pro-inflammatory tumor microenvironment, which may contribute to tumor growth and metastasis.^[21]^ Therefore, NLR, as a simple and feasible blood marker, has potential clinical application value, helping clinicians better assess the prognosis of breast cancer patients and formulate more personalized treatment strategies.

In another observational study, the prognostic significance of the platelet-to-lymphocyte ratio (PLR) in breast cancer patients was assessed. This study involved over 1000 breast cancer patients and examined the relationship between PLR and survival outcomes. The findings revealed that an elevated PLR was strongly correlated with reduced overall survival and disease-free survival, particularly in patients with metastatic breast cancer, where the high PLR group had significantly poorer outcomes compared to the low PLR group. Multivariate analysis confirmed that high PLR was an independent predictor of unfavorable prognosis. The study suggested that elevated PLR may indicate inflammation associated with tumor progression and reflect the pro-tumor activity of platelets, which could contribute to tumor growth and metastasis.^[22]^ Therefore, PLR, as a simple and feasible blood marker, has potential clinical application value, helping clinicians better assess the prognosis of breast cancer patients and formulate more personalized treatment strategies, particularly in high-risk metastatic breast cancer patients.

### 5.1. Neutrophil perturbation response (side fluorescence standard deviation of neutrophil 4 in response to alhydrogel perturbation measured by WDF dye)

Neutrophil perturbation response refers to the way neutrophils, a type of white blood cell, react to specific stimuli or disturbances. In this context, the focus is on the side fluorescence standard deviation (SD) of neutrophil 4 when exposed to alhydrogel perturbation, as measured by WDF dye. Neutrophils play a critical role in the innate immune system, acting as the first line of defense against infections by rapidly migrating to infection sites to phagocytize and neutralize pathogens. Alhydrogel, an adjuvant used to boost the immune response, is employed here to stimulate neutrophils and observe their reaction. WDF dye, a specialized fluorescent marker, is used in flow cytometry to bind to specific neutrophil components, allowing for the measurement of cell characteristics such as size, granularity, and fluorescence intensity. Side fluorescence refers to the emitted light from neutrophils when they are exposed to a laser in flow cytometry, and the SD quantifies the variability in fluorescence intensity, reflecting how neutrophils respond to the alhydrogel stimulus. Neutrophil 4 likely refers to a distinct subset of neutrophils, defined by particular surface markers or functional traits. A higher side fluorescence SD suggests greater variation in the neutrophil response to alhydrogel, while a lower SD indicates a more uniform response. Understanding how neutrophils react to various stimuli provides valuable insights into immune function and dysfunction, which is essential for developing therapies for infections, inflammatory disorders, and immune-related diseases.^[23,24]^

In breast cancer, the neutrophil perturbation response (measured by the side fluorescence standard deviation of neutrophil 4 following alhydrogel stimulation with WDF dye) may act as a protective factor through several mechanisms. Firstly, a higher side fluorescence SD in neutrophil 4 suggests enhanced immune activation, boosting the anti-tumor response by stimulating T cells and NK cells. Additionally, these neutrophils may exhibit increased cytotoxicity, releasing reactive oxygen species (ROS) and enzymes like elastase and lysozyme to directly kill tumor cells. Furthermore, neutrophils stimulated by alhydrogel can inhibit tumor angiogenesis by releasing factors like platelet factor 4 and tumor necrosis factor-related apoptosis-inducing ligand (TRAIL), thus limiting blood and nutrient supply to the tumor and preventing its growth and spread. The changes in side fluorescence SD also reflect neutrophil modulation of the tumor microenvironment, where the cytokines and chemokines they release, such as interleukin-8 (IL-8) and tumor necrosis factor-α (TNF-α), can promote local inflammation, making the environment less conducive to tumor survival. Additionally, neutrophils 4 can induce apoptosis in tumor cells through TRAIL and granzyme B, further inhibiting tumor growth. Finally, the altered side fluorescence SD may contribute to long-term immune surveillance by facilitating interactions between neutrophils, dendritic cells, and T cells, helping to form immune memory that can recognize and target tumor cells in the future. Together, these responses suggest that neutrophils play a crucial role in inhibiting breast cancer progression and metastasis.^[25–28]^ This formation of immune memory helps prevent tumor recurrence and metastasis.

Overall, this blood cell phenotype may act as a protective factor in breast cancer through potential mechanisms such as enhancing anti-tumor immune response, direct cytotoxicity, inhibition of tumor angiogenesis, regulation of tumor microenvironment, induction of tumor cell apoptosis, and promotion of immune memory.

### 5.2. Neutrophil perturbation response (forward scatter median of neutrophil 4 in response to Pam3CSK4 perturbation measured by WDF dye)

The neutrophil perturbation response, specifically the forward scatter median of neutrophil 4 after Pam3CSK4 stimulation, is used to evaluate how neutrophils (a type of white blood cell) react to specific stimuli. In this context, Pam3CSK4, a triacylated lipopeptide that activates immune responses via TLR2 (toll-like receptor 2), is employed to simulate bacterial infection. The forward scatter median measures the intensity of light scattered in the forward direction by neutrophils as they pass through a laser in a flow cytometer. This parameter is indicative of changes in cell size and internal structure, which are often associated with immune activation. The WDF dye used in the analysis binds to components of neutrophils, enabling the detection of various properties like cell size, granularity, and fluorescence intensity. A higher forward scatter median suggests that neutrophils undergo significant changes in size or internal organization, possibly indicating a stronger immune response to Pam3CSK4. Conversely, a lower forward scatter median reflects a less pronounced response with minimal alterations in neutrophil size or structure. Neutrophil 4 refers to a distinct subset of neutrophils characterized by specific markers or functions, which are analyzed to understand their specific role in immune responses.^[28–30]^

In breast cancer, the neutrophil perturbation response, reflected by the forward scatter median of neutrophil 4 in response to Pam3CSK4 perturbation, may act as a potential risk factor through several mechanisms. The increase in forward scatter median following Pam3CSK4 stimulation suggests heightened neutrophil activity, leading to the secretion of cytokines and chemokines like TNF, IL-6, and IL-8, which promote tumor cell proliferation, invasion, and metastasis. Additionally, neutrophils can facilitate angiogenesis by releasing vascular endothelial growth factor and matrix metalloproteinases (MMPs), providing nutrients and oxygen to tumors, thus supporting their growth and spread. Hyperactive neutrophils may also suppress anti-tumor immune responses by secreting IL-10 and transforming growth factor-beta, which inhibit the function of T cells and NK cells, allowing tumor cells to evade immune surveillance. Furthermore, neutrophils can recruit immunosuppressive cells such as myeloid-derived suppressor cells and regulatory T cells, enhancing the immunosuppressive tumor microenvironment that favors tumor survival. By secreting MMPs, neutrophils promote tumor cell invasion and migration, while their release of chemokines guides tumor cells to distant sites, aiding metastasis. Lastly, neutrophils can interact with tumor cells to induce multidrug resistance genes, making the tumor more resistant to chemotherapy and radiotherapy, complicating treatment and worsening prognosis.^[31–34]^

In summary, measuring the forward scatter median of neutrophil 4 in response to Pam3CSK4 perturbation can reveal the potential mechanisms by which these cells act as risk factors in breast cancer. These mechanisms include promoting tumor growth and metastasis, promoting angiogenesis, suppressing anti-tumor immune response, promoting immunosuppressive tumor microenvironment, promoting tumor cell invasion and migration, and inducing tumor resistance.

### 5.3. White blood cell perturbation response (side scatter coefficient of variation of WBC 2 in response to nigericin perturbation measured by WNR dye)

White blood cell (WBC) perturbation response, measured by the side scatter coefficient of variation (CV) of WBC 2 in response to nigericin perturbation, is a method used to assess how WBCs react to specific stimuli. White blood cells are a vital component of the immune system, playing an essential role in defending the body against infections and foreign pathogens. Nigericin, an ionophore and antibiotic, is used in experimental settings to induce stress by altering intracellular and extracellular ion concentrations. WNR dye, a fluorescent marker in flow cytometry, binds to specific cell components, enabling the measurement of cell properties such as size, granularity, and fluorescence. Side scatter (SSC) refers to the light scattered at a right angle to the laser beam during flow cytometry, often correlating with the granularity of the cell.^[35]^ The CV, calculated as the ratio of the standard deviation to the mean, quantifies the relative variability within the data. In this case, the side scatter CV reflects the variability in the internal granularity of WBCs following nigericin stimulation. White blood cell 2 refers to a specific subset of WBCs characterized by unique surface markers or functional traits. A higher side scatter CV suggests a more diverse response of WBCs, with significant changes in granularity and a heterogeneous reaction, while a lower CV indicates a more uniform response with smaller fluctuations in granularity.^[36,37]^

In breast cancer, the response of white blood cells (WBCs) to perturbations, specifically measured through the side scatter coefficient of variation (CV) of WBC 2 in response to nigericin, may play a protective role through various mechanisms: A higher CV in response to nigericin indicates a more varied and vigorous activation of white blood cells. This enhanced immune response can result in the secretion of cytokines and chemokines that recruit and activate other immune cells, such as T cells and natural killer (NK) cells, which collectively strengthen the body’s defense against tumor growth; Activated white blood cells 2 can release immune factors like TNF and interferon-gamma, which directly inhibit tumor cell proliferation, survival, and induce cell death (apoptosis). These cytokines contribute to the suppression of tumor growth and limit its progression; Upon nigericin stimulation, white blood cells 2 secrete a range of cytokines and chemokines that alter the tumor microenvironment, making it less favorable for tumor cells to thrive and spread. This includes promoting local inflammation, which further curtails tumor expansion and metastasis; White blood cells 2 can trigger apoptosis in tumor cells by releasing TRAIL and granzyme B. This process activates apoptotic pathways in tumor cells, leading to their death and hindering further tumor progression; Activated white blood cells 2 can produce factors that disrupt angiogenesis, thereby limiting the formation of new blood vessels that tumors rely on for nutrients and oxygen. By suppressing angiogenesis, the growth and spread of the tumor are restricted; Through their interactions with dendritic cells and T cells, white blood cells 2 can help establish immune memory, enhancing the body’s ability to recognize and destroy tumor cells if encountered again. This immune memory function plays a key role in preventing tumor recurrence and metastasis, providing long-term protection against breast cancer.^[38–41]^

Overall, measuring the side scatter coefficient of variation of white blood cells 2 in response to nigericin perturbation can reveal the potential protective mechanisms of these cells in breast cancer. These mechanisms include enhancing the anti-tumor immune response, inhibiting tumor growth, regulating the tumor microenvironment, promoting tumor cell apoptosis, inhibiting tumor angiogenesis, and enhancing immune memory.

### 5.4. White blood cell perturbation response (forward scatter coefficient of variation of WBC in response to Pam3CSK4 perturbation measured by WNR dye)

White blood cell perturbation response (measured by the forward scatter coefficient of variation of WBC in response to Pam3CSK4 perturbation using WNR dye) is employed to examine how white blood cells (WBCs) react to specific stimuli or perturbations. In this case, we focus on the variation in forward scatter (FSC) as a response to Pam3CSK4 exposure. White blood cells are vital components of the immune system, tasked with defending the body against infections and foreign pathogens. These cells are essential in immune responses, quickly responding to and eliminating invading microorganisms. Pam3CSK4, a triacylated lipopeptide and TLR2 (toll-like receptor 2) ligand, mimics bacterial infection and triggers immune activation. WNR dye is a fluorescent marker used in flow cytometry to label components of white blood cells, enabling the analysis of characteristics like cell size, internal granularity, and fluorescence intensity. Forward scatter (FSC) indicates the amount of light scattered in the forward direction when cells pass through a laser beam in a flow cytometer, which correlates with cell size and refractive index. The coefficient of variation (CV), defined as the ratio of the standard deviation to the mean, is a measure of data variability. In this context, the forward scatter CV quantifies the variability in the size and refractive index of white blood cells after exposure to Pam3CSK4, offering insights into the diversity of their response. A higher forward scatter CV suggests a more variable or heterogeneous response, possibly reflecting significant changes in cell size and internal structure. Conversely, a lower forward scatter CV points to a more uniform response with smaller variations in cell characteristics.^[42]^

The perturbation response of white blood cells (WBCs), measured by the forward scatter coefficient of variation (CV) in response to Pam3CSK4 stimulation using WNR dye, may act as a risk factor in breast cancer through several potential mechanisms. First, an increase in forward scatter CV after Pam3CSK4 stimulation suggests WBC hyperactivity, which can lead to the release of cytokines and chemokines like TNF, IL-6, and IL-8, promoting tumor cell proliferation, invasion, and metastasis. Second, WBCs can enhance angiogenesis by secreting vascular endothelial growth factor and MMPs, fostering the formation of new blood vessels that supply nutrients and oxygen to tumors, thereby facilitating their growth and spread. Third, hyperactive WBCs can suppress the function of other immune cells, such as T cells and NK cells, through the secretion of inhibitory cytokines like IL-10 and transforming growth factor-beta, weakening the body’s anti-tumor immune response and allowing tumor cells to escape immune surveillance. Fourth, WBCs can recruit and activate immunosuppressive cells like myeloid-derived suppressor cells and regulatory T cells, further reinforcing an immunosuppressive tumor microenvironment that promotes tumor survival. Additionally, WBC-secreted MMPs can degrade the extracellular matrix, aiding in tumor cell invasion and migration, while chemokines attract tumor cells to new sites, facilitating metastasis. Lastly, interactions between WBCs and tumor cells may induce multidrug resistance genes, making tumors more resistant to chemotherapy and radiotherapy, and worsening the prognosis. These mechanisms collectively demonstrate how perturbation responses of white blood cells contribute to the progression and spread of breast cancer.^[43,44]^

In summary, measuring the forward scatter coefficient of variation of white blood cells in response to Pam3CSK4 perturbation can reveal the potential mechanisms by which these cells act as risk factors in breast cancer. These mechanisms include promoting tumor growth and metastasis, promoting angiogenesis, suppressing anti-tumor immune response, promoting immunosuppressive tumor microenvironment, promoting tumor cell invasion and migration, and inducing tumor resistance.^[45,46]^

This research highlights 2 specific blood cell phenotypes that may act as protective factors against breast cancer, suggesting their potential in hindering tumor growth or enhancing immune defense against cancer cells. Conversely, 2 other blood cell phenotypes were found to be risk factors, potentially heightening breast cancer risk by supporting tumor progression or suppressing immune function. By uncovering the links between these blood cell phenotypes and breast cancer, this study not only advances our understanding of the disease’s pathological mechanisms but also lays a solid foundation for future preclinical studies and clinical therapies, offering crucial insights for the development of novel prevention and treatment strategies.

The implications of these findings are far-reaching, as they provide vital theoretical support for future animal experiments and clinical applications. These results deepen our comprehension of the biological underpinnings of breast cancer and may inspire new strategies for prevention and therapy. By targeting these blood cell phenotypes, more effective interventions could be developed to enhance the management of breast cancer and improve treatment outcomes. Additionally, these findings open up new possibilities for personalized medicine, enabling treatments tailored to an individual’s specific blood cell profile, which could increase treatment efficacy while minimizing adverse effects.

In preclinical settings, these results can inform the design of more precise experiments to confirm the roles of these blood cell phenotypes in cancer progression and explore strategies to modulate them to disrupt tumor growth. Clinically, this research can help identify at-risk individuals, facilitating early detection and preventive measures. Moreover, a deeper exploration of the molecular mechanisms behind these phenotypes could reveal new drug targets, contributing to the advancement of novel anti-cancer therapies.

Overall, this study not only provides new perspectives for basic research on breast cancer but also offers valuable information for early diagnosis, personalized treatment, and prevention strategies in clinical practice, potentially significantly improving the survival rate and quality of life of breast cancer patients.

The significance of this study lies in its systematic and comprehensive exploration of the potential causal relationships between blood cell phenotypes and breast cancer, which is crucial for deepening the understanding of the mechanisms underlying breast cancer. The study employed a 2-sample MR approach, leveraging genetic variants as instrumental variables to effectively overcome biases caused by confounding factors and reverse causation in traditional observational studies. This allowed for a more precise investigation of the causal effects of hematological traits on the development and progression of breast cancer.

By analyzing the associations between 91 blood cell phenotypes and breast cancer and validating the findings using data from 3 independent sources, the study improved the generalizability and external validity of the results. Subsequently, a meta-analysis was conducted to integrate the primary results, further enhancing statistical power and robustness. Additionally, multiple testing corrections were applied to ensure the reliability and accuracy of the findings. Importantly, for blood cell phenotypes showing significant associations, reverse causation analyses were conducted to determine the direction of causality, thereby increasing the credibility of causal inferences.

The findings of this study provide important insights into the pathobiology of breast cancer, suggesting that certain blood phenotypes may not only serve as potential biomarkers but also play a role in its pathophysiological processes. This contributes to a better understanding of breast cancer etiology and offers a scientific basis for the development of early prediction tools and precision therapeutic strategies. Furthermore, the innovative and rigorous methodological approach serves as a valuable example for studying similar diseases, offering significant academic value and potential for clinical translation.

Based on multiple observational studies, this study revealed the causal relationship between blood cell phenotypes and breast cancer through genetic-level validation, achieving a completely randomized controlled trial at the genetic level. This method avoids confounding factors in observational studies and more accurately elucidates the relationship between the 2. By combining MR analysis and meta-analysis, the results are more reliable and credible. However, the study also has some limitations. Since the data mainly comes from European populations, the results may not fully represent the global population. Future research should extend to other ethnicities and regions to further validate and enrich these findings.

## 6. Conclusions

This study used a bidirectional MR combined with meta-analysis to verify the causal relationship between blood cell phenotypes and breast cancer. The evidence shows that the blood cell phenotypes Neutrophil perturbation response (side fluorescence standard deviation of neutrophil 4 in response to alhydrogel perturbation measured by WDF dye) and White blood cell perturbation response (side scatter coefficient of variation of WBC 2 in response to nigericin perturbation measured by WNR dye) are protective factors for breast cancer. In contrast, the blood cell phenotypes Neutrophil perturbation response (forward scatter median of neutrophil 4 in response to Pam3CSK4 perturbation measured by WDF dye) and White blood cell perturbation response (forward scatter coefficient of variation of WBC in response to Pam3CSK4 perturbation measured by WNR dye) are risk factors for breast cancer. Moreover, there is no reverse causal relationship between these 4 blood cell phenotypes and breast cancer.

By monitoring these specific blood cell phenotypes, doctors can identify high-risk individuals earlier and take preventive measures. This blood cell phenotype-based risk assessment method is noninvasive and highly operational, making it suitable for implementation during routine checkups. Understanding the relationship between these blood cell phenotypes and breast cancer risk can help develop personalized treatment and prevention strategies. For instance, individuals with high-risk blood cell phenotypes may be considered for more frequent screenings or preventive measures, while those with protective phenotypes may avoid overtreatment.

The study’s findings provide new clues for further exploring the pathological mechanisms of breast cancer. Understanding how these blood cell phenotypes influence the development of breast cancer can drive the development of new therapeutic methods, such as treatments targeting blood cell phenotypes. Additionally, the results can guide public health policy by promoting blood cell phenotype-based screening methods in high-risk populations, improving early detection and treatment outcomes for breast cancer, thus reducing the incidence and mortality rates of breast cancer.

## Acknowledgments

Firstly, we express our profound thanks to all individuals and researchers who participated in the GWAS data for this research. Additionally, we extend our sincere gratitude and respect to the personnel involved with the associated public databases. Lastly, our heartfelt appreciation goes out to every author who played a role in contributing to this study.

## Author contributions

**Conceptualization:** Qi Liu, Yi Zhang, Lin Yang, Dongdong Wan.

**Data curation:** Wei Jia, Yi Zhang, Jun Lu, Qingbin Luo.

**Formal analysis:** Qi Liu, Yi Zhang, Jun Lu, Lin Yang.

**Investigation:** Qi Liu, Wei Jia, Yi Zhang, Jun Lu, Qingbin Luo.

**Methodology:** Qi Liu, Wei Jia, Yi Zhang, Jun Lu, Qingbin Luo, Lin Yang, Dongdong Wan.

**Project administration:** Qi Liu, Wei Jia, Yi Zhang, Jun Lu, Qingbin Luo, Lin Yang, Dongdong Wan.

**Resources:** Dongdong Wan.

**Software:** Qi Liu, Wei Jia.

**Supervision:** Lin Yang, Dongdong Wan.

**Validation:** Qingbin Luo, Lin Yang, Dongdong Wan.

**Visualization:** Qi Liu, Wei Jia.

**Writing – original draft:** Qi Liu, Wei Jia, Yi Zhang, Jun Lu.

**Writing – review & editing:** Lin Yang, Dongdong Wan.

## Supplementary Material


